# Targeted scVEGF/^177^Lu radiopharmaceutical inhibits growth of metastases and can be effectively combined with chemotherapy

**DOI:** 10.1186/s13550-016-0163-1

**Published:** 2016-01-16

**Authors:** Mary Rusckowski, Yuzhen Wang, Francis G. Blankenberg, Zoia Levashova, Marina V. Backer, Joseph M. Backer

**Affiliations:** Department of Radiology, University of Massachusetts Medical School, Worcester, MA 01655 USA; Department of Radiology/MIPS, Stanford University, Palo Alto, CA 94305 USA; Current address: Igenica Biotherapeutics, Inc., Burlingame, CA 94010 USA; Sibtech, Inc., Brookfield, CT 06804 USA

**Keywords:** VEGF receptors, ^177^Lu, Targeted radiopharmaceutical, Breast cancer, Metastases

## Abstract

**Background:**

scVEGF/^177^Lu is a novel radiopharmaceutical targeted by recombinant single-chain (sc) derivative of vascular endothelial growth factor (VEGF) that binds to and is internalized by vascular endothelial growth factor receptors (VEGFR). scVEGF/^177^Lu potential as adjuvant and neoadjuvant anti-angiogenic therapy was assessed in metastatic and orthotopic mouse models of triple-negative breast cancer.

**Methods:**

Metastatic lesions in Balb/c mice were established by intracardiac injection of luciferase-expressing 4T1luc mouse breast carcinoma cells. Mice with metastatic lesions received single intravenous (i.v.) injection of well-tolerated dose of scVEGF/^177^Lu (7.4 MBq/mouse) at day 8 after 4T1luc cell injection. Primary orthotopic breast tumors in immunodeficient mice were established by injecting luciferase-expressing MDA231luc human breast carcinoma cells into mammary fat pad. Tumor-bearing mice were treated with single injections of scVEGF/^177^Lu (7.4 MBq/mouse, i.v), or liposomal doxorubicin (Doxil, 1 mg doxorubicin per kg, i.v.), or with a combination of Doxil and scVEGF/^177^Lu given at the same doses, but two hours apart. “Cold” scVEGF-targeting conjugate was included in controls and in Doxil alone group. The effects of treatments were defined by bioluminescent imaging (BLI), computed tomography (CT), computed microtomography (microCT), measurements of primary tumor growth, and immunohistochemical analysis.

**Results:**

In metastatic model, adjuvant treatment with scVEGF/^177^Lu decreased overall metastatic burden and improved survival. In orthotopic primary tumor model, a combination of Doxil and scVEGF/^177^Lu was more efficient in tumor growth inhibition than each treatment alone. scVEGF/^177^Lu treatment decreased immunostaining for VEGFR-1, VEGFR-2, and pro-tumorigenic M2-type macrophage marker CD206.

**Conclusions:**

Selective targeting of VEGFR with well-tolerated doses of scVEGF/^177^Lu is effective in metastatic and primary breast cancer models and can be combined with chemotherapy. As high level of VEGFR expression is a common feature in a variety of cancers, targeted delivery of ^177^Lu for specific receptor-mediated uptake warrants further exploration.

**Electronic supplementary material:**

The online version of this article (doi:10.1186/s13550-016-0163-1) contains supplementary material, which is available to authorized users.

## Background

Targeting VEGF signaling via VEGF receptors in tumor vasculature has become a well-established treatment modality, known as anti-angiogenic therapy [1–5]. Several major anti-angiogenic drugs are already in the clinic, and a multitude of clinical trials for various cancers are in progress [1]. Unfortunately, these inhibitors are, at best, only marginally effective and, in many cases, are not effective at all, as was recently underscored by the failures in phase III breast cancer clinical trials [6]. The current explanation for this ineffectiveness is that VEGF/VEGFR inhibitors induce only a transient regression of tumor vasculature followed by vascular rebound that rekindles aggressive tumor growth [7–9]. Two mechanisms are, apparently, involved in vascular rebound: (1) so-called evasive resistance of endothelial cells, which allows them to use pathways other than VEGF/VEGFR for growth and survival, and (2) only recently appreciated systemic pro-angiogenic/pro-inflammatory/pro-tumorigenic host responses induced by inhibition of VEGF/VEGFR signaling in VEGFR-positive cell populations, including endothelial, immune, progenitor, and stem cells [10–21].

With the goal of enhancing the efficacy of anti-angiogenic therapy, we are developing a novel radiopharmaceutical that is designed for VEGFR-mediated intracellular delivery of ^177^Lu [22]. We reasoned that since such a radiopharmaceutical is not an inhibitor of VEGFR signaling, it would not lead to either evasive resistance of endothelial cells or to systemic responses associated with inhibition of this pathway in various host cells. On the other hand, we expected that VEGFR-mediated intracellular ^177^Lu accumulation would lead to a dose-dependent radiotoxicity, which would be more pronounced in VEGFR-overexpressing tumor endothelial cells relative to the cells with physiologically normal levels of VEGFR. Critically, particularly high levels of VEGFRs are found in tumor endothelial cells that are positioned at the tips of growing angiogenic vascular sprouts [23], making them the primary targets for scVEGF/^177^Lu.

To enable VEGFR-mediated delivery, we employed scVEGF-PEG-DOTA targeting conjugate, which is a derivative of human VEGF-A re-engineered into a single-chain polypeptide format (scVEGF), and site-specifically derivatized with PEGylated DOTA chelator [24]. Like parental VEGF-A, various site-specific scVEGF conjugates, including radiolabeled scVEGF-PEG-DOTA conjugates, bind to VEGFRs with high affinity and undergo VEGFR-mediated internalization [22, 24].

We initially tested ^177^Lu-radiolabeled scVEGF-PEG-DOTA (scVEGF/^177^Lu) in an orthotopic triple-negative breast cancer model in syngeneic and immunocompromised mice [22]. We focused on breast cancer because current anti-angiogenic drugs are particularly inefficient for this disease [6]. Biodistribution, radiotoxicity, dosimetry, and dose-dependence studies established a safe and well-tolerated dose for a single i.v. injection of scVEGF/^177^Lu that led to significant tumor growth inhibition [22], with tumor uptake (2.32 + 0.14 %ID/g) similar to that for PET and SPECT tracers based on scVEGF-PEG-DOTA conjugate [24]. The most remarkable outcome of single scVEGF/^177^Lu injection was a long-term sustainable vascular regression and, particularly, the decline in the prevalence of VEGFR-2 overexpressing cells in tumor vasculature; the effects were only transient in mice treated with conventional anti-angiogenic VEGF/VEGFR inhibitors, bevacizumab, or sunitinib [22]. Interestingly, combining scVEGF/^177^Lu with these anti-angiogenic drugs affected only a subset of mice, increasing the fraction of mice with slow growing tumors [22]. These findings indicated that scVEGF/^177^Lu might be a more effective drug than the current inhibitors of VEGFR signaling and stimulated a further exploration of scVEGF/^177^Lu in more realistic models of disease and treatment.

In this study, we explored a possibility of incorporating scVEGF/^177^Lu into current therapy for breast cancer, considering both adjuvant and neoadjuvant settings. The majority of breast cancer patients undergo adjuvant therapy, because local and distant recurrent disease is frequent, with metastatic dissemination being the main cause of mortality. As a relevant model for adjuvant therapy, we selected an aggressive and highly metastatic 4T1 murine breast carcinoma grown in immunocompetent Balb/c mice. We report here that single well-tolerated dose of scVEGF/^177^Lu is effective in inhibiting metastatic growth of 4T1luc in Balb/c mice that warrants a further development of this radiopharmaceutical as a stand-alone or combination adjuvant therapy to prevent metastatic dissemination in post-surgery breast cancer patients.

Improving the outcome of neoadjuvant therapy is an unmet clinical need, which is particularly important for triple-negative breast cancer (TNBC) patients because of so-called triple-negative paradox [25]. TNBC patients have higher rates of pathological complete response to neoadjuvant therapy than patients with other breast tumor types. However, those who do not respond have a significantly worse prognosis [25]. Thus, improving neoadjuvant therapy with timely incorporation of scVEGF/^177^Lu treatment may have a significant clinical impact. We report here that scVEGF/^177^Lu can be efficiently combined with doxorubicin chemotherapy, a standard front-line chemotherapeutic for breast cancer, in a model of orthotopic human MDA231luc xenograft, suggesting a potential application as a neoadjuvant combination therapy for breast cancer.

## Methods

### Cells and reagents

MDA231luc cells are derivatives of MDA-MB-231 human breast cancer cells engineered to stably express firefly luciferase [24]. 4T1luc cells are derivatives of 4T1 mouse mammary tumor cells engineered to stably express firefly luciferase [24]. Both cell lines were cultured in Dulbecco’s modified Eagle medium (DMEM) media (Gibco) with non-essential amino acids, 10 % fetal bovine serum, and 1 % penicillin/streptomycin at 37 °C in a humidified atmosphere with 5 % CO_2_. Lutetium-177 chloride was from PerkinElmer Life Sciences Inc. (Boston, MA). The liposomal formulation of doxorubicin (Doxil) was from Janssen Products, LP (Horsham, PA). scVEGF-PEG-DOTA was produced at SibTech, as described [24]. All other chemicals were analytical grade from standard suppliers.

### Synthesis of scVEGF-PEG-DOTA

Preparation of scVEGF-PEG-DOTA conjugate followed [24] included some modifications of the published protocol [24]. We first conjugated 3.4 kDa bifunctional maleimide-PEG-amine to NHS-DOTA chelator via NHS chemistry, as opposed to using maleimide-PEG-NHS and DOTA-Bz-amine described in [24], and then site-specifically conjugating maleimide-PEG-DOTA to thiol group in Cys-tag of scVEGF via maleimide chemistry.

### Radiolabeling scVEGF-PEG-DOTA with ^177^Lu

Radiolabeling was performed as described [22] with minor modifications. Briefly, ^177^LuCl_3_ in 0.05 M HCl (about 480 MBq, 9 μl) was added to 0.1 M sodium acetate (40 μl), pH 5.5, for a final pH of 5.0, transferred to scVEGF-PEG-DOTA (150 μg, 70 μl) in 0.1 M sodium acetate, pH 5.5, and heated at 55 °C for 1 h. The labeling reaction was loaded on a PD-10 column equilibrated with 0.1 % BSA in phosphate-buffered saline (PBS) and scVEGF/^177^Lu fractions were collected. Labeling efficiency and radiochemical purity was determined by TLC on Whatman No. 1 paper with saturated sodium chloride as solvent, a system where scVEGF/^177^Lu is retain at start, while free ^177^Lu has *R*_*f*_ > 0.6. Radiolabeled conjugate was also characterized by HPLC on a Superose™ 12 10/300 column eluted with 0.6 mL/min 80 % 0.1 M Tris-HCl, pH 8.0 and 20 % acetonitrile, where scVEGF/^177^Lu is eluted at ~13 min, while free ^177^Lu is eluted 25–29 min. The labeling efficiency by HPLC was about 80–85 %, and by TLC was 99 %, and specific activity was in the range of 2.2–2.4 MBq/μg.

### Animal experiments

All animal experiments were approved by the Institutional Animal Care and Use Committee of the University of Massachusetts Medical School (Worcester, MA) and Stanford University (Palo Alto, CA). Female BALB/c and SCID mice (Charles River Laboratories International, Inc., Wilmington, MA), weighing 17 to 22 g, about 4–6 weeks old, were fed standard mouse chow and water, and housed under standard conditions.

To establish metastatic breast cancer model, Balb/c mice were inoculated with 4T1luc cells via intracardiac injection. 4T1luc cells were grown to 70–80 % of confluence in T-75 flasks, treated with ×1 trypsin/EDTA, washed, and suspended in Dulbecco’s phosphate-buffered saline (DPBS) at 5 × 10^5^ per milliliter. The cells were kept on ice and delivery was completed within 1 h after harvesting. Animals were anesthetized with isoflurane and placed on a sterile drape with limbs secured and chest swabbed with 70 % ethanol. A 0.3-cm^3^ syringe (29G needle) containing 5 × 10^4^ 4T1luc cells in 0.1 ml DPBS was inserted slowly into the chest in the second intercostal space directly into the left ventricle. Proper placement in the left ventricle was indicated by blood flow into the syringe, and the cells were delivered slowly (approximately 30 s for 0.1 ml). Once complete, the needle was withdrawn by pulling straight out quickly, and the animal was placed on a heating pad and monitored until awake and ambulatory.

To establish MDA231luc orthotopic breast cancer model, immunodeficient SCID mice were inoculated with MDA231luc cells into mammary fat pads. MDA231luc cells were grown to 90 % of confluence in T-75 flasks, treated with ×1 trypsin/EDTA, washed, and suspended in cold serum-free DMEM at 80 × 10^6^ cells per milliliter. The cells were kept on ice, and delivery was completed within 1 h of harvest. For inoculation, the animals were anesthetized with isoflurane and cells were resuspended by gentle pipetting before drawing 50 μl (4 × 10^6^ cells) into a 0.5-ml syringe (28G needle). The needle with bevel up was inserted into the fourth mammary fat pad, and cells delivered slowly. The same was repeated on the opposite side, resulting in two tumors per mouse. After injection, the mice were kept sedated on a heating pad for about 40 min.

### Monitoring and treatment of 4T1luc metastatic lesions

4T1luc spread and growth were monitored by whole body bioluminescent imaging (BLI) in ventral and dorsal positions. BLI was acquired on an IVIS 100 (PerkinElmer) with exposure of 60 s about 12–15 min after intraperitoneal luciferin injection (PerkinElmer, Waltham, MA), at dose of 150 mg/kg of body weight. Whole body bioluminescence was measured by Living Image 4.1 software using a sum of both dorsal and ventral views. Bone lesions were documented with microtomography (microCT). Baseline scans were acquired prior to cell delivery on day 0 and then on day 4 and at 2–3 day intervals thereafter. The CT was acquired on a small animal NanoSPECT/CT with 65 kVp voltage and 500 ms exposure time, and the CT reconstructions were with InVivoScope 1.43 software (Bioscan, Washington, DC). Bone metastasis was also confirmed on the final day by high-resolution microCT on a SCANCO vivaCT 75 microCT with 20.5 μm scan resolution (Scanco USA, Inc., Wayne, PA).

On day 8 after cell inoculation, ten mice with leg bone metastasis confirmed by BLI received scVEGF/^177^Lu (5–6 μg protein/mouse, 7.4 MBq/mouse) in 50 μl via retro-orbital sinus injection. Seven mice received equivalent amounts of unlabeled scVEGF-PEG-DOTA conjugate as control. On days 15–17, the animals were euthanized; leg bones and visceral metastases were harvested for analysis.

### Treatment of MDA231 orthotopic tumors

Following MDA231luc cells inoculation, mice were observed daily and tumors were measured by calipers every 2–3 days. Tumor volume was calculated as *V* = 0.5*(L*W*H), where *L* is the long axis, *W* is the short axis, and *H* is the height of a tumor.

When the tumors reached 50–200 mm^3^, about 2–3 weeks after tumor cell inoculation, animals were randomized into four treatment groups: (1) unlabeled scVEGF-PEG-DOTA, (*n* = 20); (2) scVEGF/^177^Lu (7.4 MBq/mouse, *n* = 10); (3) Doxil plus unlabeled scVEGF-PEG-DOTA, (*n* = 9); and (4) Doxil plus scVEGF/^177^Lu (7.4 MBq/mouse, *n* = 15). scVEGF/^177^Lu or scVEGF-PEG-DOTA (5–6 μg/mouse) were delivered in 50 μl via retro-orbital sinus injection and a single dose of Doxil (1 mg/kg body weight) was delivered in 100 μl via tail vein injection.

Animals were euthanized between 7 and 14 days after treatment. Tumors were harvested and snap-frozen in OCT for immunohistochemical analysis (IHC).

### IHC

Bone samples from both hind legs in the 4T1luc metastatic model were fixed in 10 % formalin for 48 h, rinsed in PBS, decalcified in 14 % EDTA solution for 10–14 days, and embedded in paraffin. To unmask biomarkers, 5-μm sections were de-waxed and incubated in 10 mM sodium citrate pH 6, 0.05 % Tween-20 at 95 °C for 30 min. Double fluorescent staining with tyramine amplification technique was done as described in details recently [26], with the following primary antibodies. For formalin-fixed tissue, VEGFR-2-specific (Cell Signaling #2479BC), VEGFR-1-specific (Abcam C-terminal-specific #Ab2350), and CD31-specific (DiaNova #DIA-310-M); for snap-frozen primary tumors and visceral metastases, CD31-specific and VEGFR-2-specific antibodies (BD Pharmingen #550274 and #550549) and CD206-specific antibody (Serotek #1111) were used. Slides were mounted in medium for fluorescence with DAPI for nuclear counterstaining (Vector Laboratories) and observed in Zeiss Axiovert microscope. For quantitative analyses of immunofluorescent staining, digital images of multiple microscopic fields were obtained under identical conditions, and the percentage of pixels containing antigen-specific staining above the same threshold for a selected region of interest was determined by histogram analysis using Zeiss software.

## Results

### Single injection of scVEGF/^177^Lu inhibits growth of metastases

**Fig. 1:**
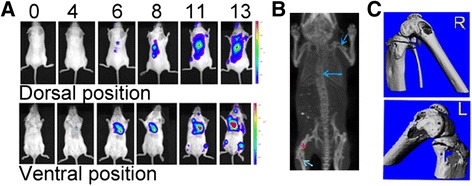
Growth of 4T1luc mouse breast carcinoma metastatic lesions in syngeneic Balb/c mice. **a** Representative longitudinal whole body BLI performed during 13 days after intracardiac injection of 4T1luc cells. Prior to imaging mice received lucefirin (0.5 mg/mouse, intraperitonially). **b** Representative CT imaging of metastatic bone lesions at day 13 after 4T1luc injection. *Blue arrows* indicate metastatic lesions. *Red cross* marks left hind leg. **c** MicroCT image of right and left hind leg knee. Areas of significant pitting and large areas of osteolysis associated with bone metastatic lesions are visible around knee joints

**Fig. 2:**
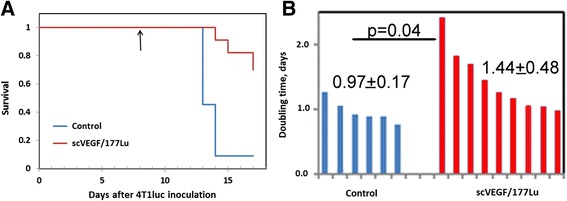
Single injection of scVEGF/^177^Lu at day 8 after 4T1luc inoculation delays metastases-induced mortality and inhibits metastatic growth. Control mice received equivalent amounts of cold scVEGF-PEG-DOTA. **a** Kaplan-Meier analysis of survival in control (*blue*) and treated (*red*) mice. **b** Doubling time values for longitudinal increase in bioluminescent intensity of total metastatic burden for individual control (*blue*) and treated (*red*) mice. Average and *p* values are indicated

To explore scVEGF/^177^Lu in adjuvant settings, we used a mouse model with metastatic lesions but without primary tumor. Intracardiac injection of 4T1luc cells in Balb/c resulted in rapid appearance of visceral and bone metastases that were readily detectable by BLI, CT, and microCT (Fig. 1a–c and Additional file 1: Figure S1 and Additional file 2: Figure S2) and confirmed by H&E staining of harvested tissue and bones (not shown). To assess effects of scVEGF/^177^Lu on progression of metastases, at day 8 after tumor cell inoculation, mice in treatment group (*n* = 11) received single injection of previously established [22] well-tolerated dose of scVEGF/^177^Lu (7.4 MBq/mouse) via retro-orbital sinus. To avoid potential confounding effects, control mice (*n* = 11) received the equivalent amounts of “cold,” unlabeled scVEGF-PEG-DOTA conjugate. Kaplan-Meier survival analysis indicated that scVEGF/^177^Lu-treated mice survived significantly better than control mice (Fig. 2a), with difference characterized by chi-squared statistics of 9.02 (*p* < 0.0015) and hazard ratio of 5.73 (5.15 to 6.31 for 95 % confidence interval).

In order to further assess the development of metastatic burden in individual mice, we performed longitudinal whole body BLI in each control and treated mouse. Since BLI after luciferine injection was due only to luciferase-expressing tumor cells, we reasoned that quantitative BLI might provide a useful comparison between treated and control mice. Indeed, longitudinal BLI changes in individual mice were readily approximated as exponential (*R*^2^ > 0.9) in all but one mouse in treated group and one mouse in control group, which allowed for calculation of BLI “doubling time” as a surrogate parameter for changes in metastatic burden. The calculated doubling time was found to be significantly longer (1.44 ± 0.48 vs. 0.97 ± 0.17 day, *p* = 0.04) for treated vs. control mice (Fig. 2b), indicating that a slower growth of metastatic burden is, most likely, responsible for the delayed mortality in scVEGF/^177^Lu-treated mice (Fig. 2a).

**Fig. 3:**
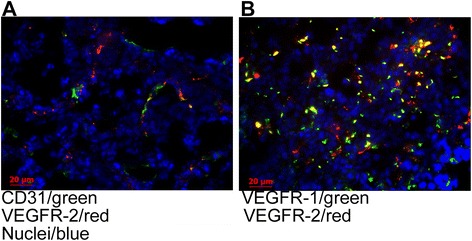
In kidney metastatic lesions, VEGFR-2 is expressed predominantly on CD31^+^ endothelial cells, while more abundant VEGFR-1 is predominantly expressed on non-endothelial cells. **a** Colocalization of immunofluorescent staining for CD31 (*green*) and VEGFR-2 (*red*). **b** VEGFR-1 (*green*) and VEGFR-2 (*red*) are expressed mostly on non-overlapping subsets of cells. Nuclei stained with DAPI (*blue*). ×40 objective

Immunofluorescent analysis of harvested visceral metastatic lesions shows that VEGFR-2 colocalized well with a pan-endothelial marker CD31, indicating that VEGFR-2 is predominantly expressed on tumor endothelium (Fig. 3a, for a kidney metastatic lesion). In contrast, in the same visceral metastases, VEGFR-1 only occasionally colocalized with CD31 (not shown) or VEGFR-2 (Fig. 3b), indicating that VEGFR-1 is predominantly expressed on non-endothelial cells.

**Fig. 4:**
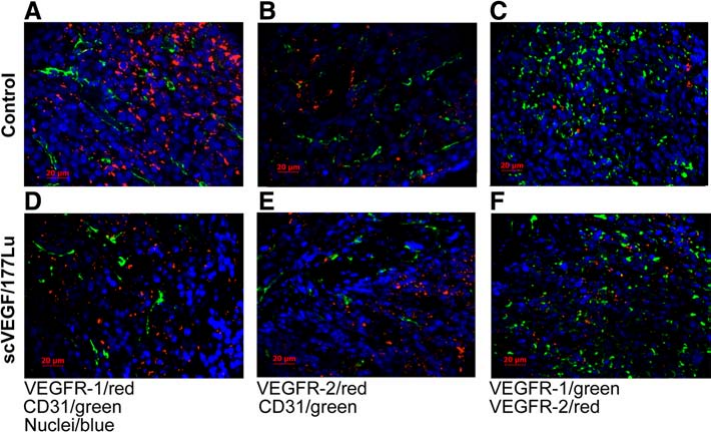
In bone metastatic lesions, VEGFR-1 and VEGFR-2 are expressed on non-overlapping subsets of non-endothelial cells. *Top panels*, Control group. *Bottom panels*, scVEGF/^177^Lu treated group. **a**, **d** Lack of colocalization between VEGFR-1 and CD31. **b**, **e** Lack of colocalization between VEGFR-2 and CD31. **c**, **f** Lack of colocalization between VEGFR-1 and VEGFR-2. Nuclei stained with DAPI (*blue*). ×40 objective

In bone metastases, VEGFR-1 was more widespread than VEGFR-2, and both receptors mostly did not colocalize with each other or with CD31, indicating that both VEGFRs are predominantly expressed on non-endothelial cells (Fig. 4a–c for control and Fig. 4d–f for scVEGF/^177^Lu-treated mice). Although VEGFR-1 immunostaining was highly heterogeneous, it appears that the prevalence of VEGFR-1 in bone metastatic lesions was decreased in scVEGF/^177^Lu-treated mice, relative to control (compare Fig. 4a, d).

### Combining scVEGF/^177^Lu with chemotherapy

To explore scVEGF/^177^Lu in neoadjuvant settings, we employed immunodeficient mouse model with orthotopic human breast MDA231luc xenografts [22, 24]. For combination therapy, we used a single injection of clinical grade doxorubicin-loaded liposomes, Doxil. We reasoned that liposomes could remain entrapped in tumor after scVEGF/^177^Lu-induced vascular regression, while free drug would have a more limited access and/or cleared faster from tumor tissue. To account for potential effects of scVEGF moiety in scVEGF/^177^Lu-treated groups, both control groups (no treatment and Doxil alone) received the equivalent doses of “cold” scVEGF-PEG-DOTA conjugate.

**Fig. 5:**
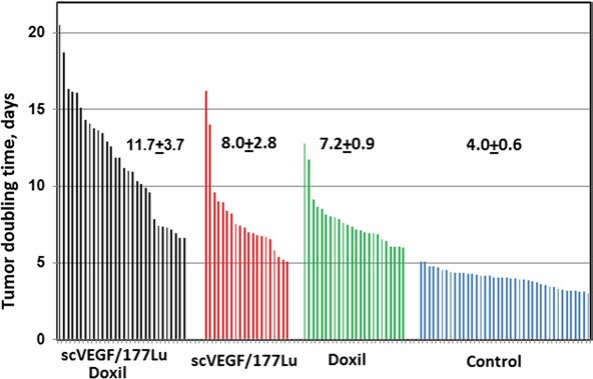
Combining scVEGF/^177^Lu with Doxil inhibits tumor growth more efficiently than single-agent treatment in orthotopic MDA231luc human breast carcinoma model in SCID mice. Effects of scVEGF/^177^Lu+Doxil, scVEGF/^177^Lu alone or Doxil (*+* equivalent amounts of cold scVEGF-PEG-DOTA), relative to control tumors injected with equivalent amounts of cold scVEGF-PEG-DOTA conjugate alone. Growth curves for individual tumors were approximated as exponential and tumor doubling time was calculated for each tumor and the sets of data presented as watershed plots for each group. Average doubling time ±STD is indicated on the plot for each group

For quantitative analysis of tumor growth, it was approximated as exponential and tumor volume doubling times were determined for individual tumors. We found a significant diversity in the rates of tumor growth in each group (Fig. 5) and even between the rates of tumor growth in the right and left mammary fat pad in the same mouse (not shown). However, for mice treated with combination therapy, the average doubling time was significantly longer than that for mice treated with either scVEGF/^177^Lu (*p* < 0.0003) or Doxil (*p* < 0.0001) alone. Of note, scVEGF/^177^Lu or a combination of scVEGF/^177^Lu with Doxil caused similar regression of tumor vasculature, as judged by VEGFR-2 prevalence on tumor cryosections, while Doxil alone did not cause any vascular regression (Additional file 3: Figure S3).

### scVEGF/^177^Lu treatment decreases CD206^+^ M2-type macrophages

**Fig. 6:**
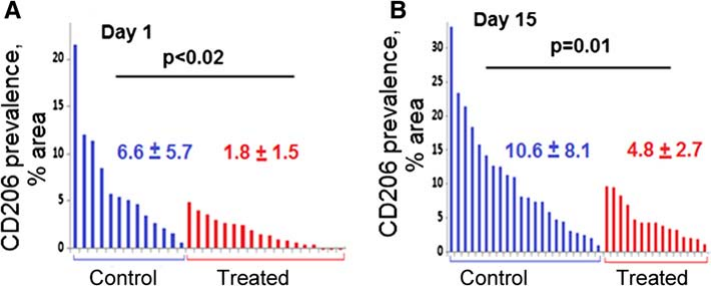
scVEGF/^177^Lu induces a decrease in CD206 immunostaining in primary orthotopic MDA231luc tumors. Images of CD206 immunostaining were captured with ×5 objective. Waterfall plots for CD206 prevalence in individual microscopic fields on immunostained cryosections for control and scVEGF/^177^Lu-treated MDA231luc tumor-bearing mice at day 1 (**a**) and day 15 (**b**) after treatment. For each group, cryosections were prepared from four tumors harvested from two mice

The effects of scVEGF/^177^Lu treatment on the prevalence of pro-tumorigenic M2-type tumor-associated macrophages (TAM) were assessed by CD206 immunostaining. Although CD206 prevalence was highly heterogeneous, we found that scVEGF/^177^Lu induced a decrease in average CD206 prevalence in tumor tissue in MDA231luc primary tumor model. This effect was detectable as early as on day 1 after treatment and was sustained at least until day 15 (Fig. 6a, b). Interestingly, this rapid CD206 decline was similar to that observed for VEGFR-2 prevalence in treated mice (Additional file 4: Figure S4). We also observed a scVEGF/^177^Lu-induced decline in CD206 prevalence in kidney metastatic lesions in 4T1luc metastatic model (Additional file 5: Figure S5).

## Discussion

Since both innate and adaptive immunity critically affect responses to anti-cancer therapy [27–29], we explored effects of adjuvant treatment with scVEGF/^177^Lu in immunocompetent mice without primary tumor but with established 4T1luc metastatic lesions (Fig. 1, Additional file 1: Figure S1 and Additional file 2: Figure S2), which typically kill mice after 12–14 days. We found (Fig. 2) that a single injection of a safe dose of scVEGF/^177^Lu resulted in rather dramatic decrease in mortality on this time scale with hazard ratio of 5.73 (5.15 to 6.31 for 95 % confidence interval). A slower growth of metastatic lesions in scVEGF/^177^Lu-treated animals, as assessed by the whole animal BLI, further supports the effectiveness of scVEGF/^177^Lu treatment.

In exploring the putative scVEGF/^177^Lu targets, VEGFR-1 and VEGFR-2, we found that VEGFR-2 prevalence was significantly higher than that of VEGFR-1 in visceral metastases, but the opposite was true for bone metastases (Figs. 3 and 4). Furthermore, in visceral metastases, only VEGFR-2 receptors were expressed predominantly on endothelial cells, as judged by colocalization with a pan-endothelial marker CD31 (Fig. 3), while in bone metastases, both VEGFR-1 and VEGFR-2 were largely expressed on different non-endothelial cells (Fig. 4).

Recent studies in tumor models and human tumors indicate that, apart from their role in tumor endothelial cells, VEGF receptors may be intimately involved in tumor cell physiology, including “stemness” of tumor cells [30, 31]. VEGFR-2 expression was reported in 4T1cells [32], while VEGFR-1 is expressed on several types of tumor cells, both in vitro and in vivo [21], including at least 16 human breast carcinoma cell lines [33, 34], and it is particularly prominent in osteosarcoma bone lesions [35]. Moreover, in a large patient cohort, VEGFR-1 expression by cancer cells in tumor biopsies was correlated with negative prognosis [36]. Our finding of widespread VEGFR-1 expression on tumor cells in bone lesions suggests that VEGFR-1 could be a prominent target for scVEGF/^177^Lu in this type of breast cancer lesions. It is also tempting to speculate that a radiopharmaceutical based on a VEGFR-1 specific mutant of scVEGF might provide additional advantages in targeting VEGFR-1-positive tumor cells in metastatic lesions, and we are currently testing this hypothesis.

Since developing scVEGF/^177^Lu as a part of combination therapy would be a more realistic translational approach, we combined it with liposomal formulation of doxorubicin (Doxil), a standard front-line chemotherapeutic for breast cancer, for treatment of orthotopic MDA231luc tumors. In regimens selected for monotherapy, either Doxil or scVEGF/^177^Lu inhibited tumor growth approximately twofold. However, a near simultaneous (only 2 h apart) treatment with both drugs effectively blocked tumor growth (Fig. 5). This is in contrast with previously tested combinations of scVEGF/^177^Lu with anti-angiogenic drugs, bevacizumab or sunitinib, which affected growth of MDA231luc tumors only in a subset of mice [22]. Taking together, our data indicate that combining scVEGF/^177^Lu low doses of front-line chemotherapy might have a higher therapeutic potential than combination with anti-angiogenic drugs.

Originally, anti-angiogenic drugs were expected to work through tumor starvation caused by vascular regression [1–6]; however, more recently systemic pro-angiogenic/pro-inflammatory/pro-tumorigenic host responses induced by these drugs are being taken into consideration as well [10–21]. One of the culprits in these responses are tumor-associated macrophages (TAMs), which are the major component of tumor stroma in invasive breast carcinomas and their enhanced accumulation is associated with poor prognosis [37, 38]. TAMs are predominantly of pro-angiogenic, anti-inflammatory, and immunosuppressive M2-type macrophages that are characterized by the production of cytokines supporting these activities [39, 40]. Typically, treatment with either anti-cancer chemotherapeutics or anti-angiogenic drugs increases the prevalence of M2-type TAMs, which blunts the response to chemotherapy through several mechanisms [39, 40]. Surprisingly, although treatment with scVEGF/^177^Lu is clearly anti-angiogenic, unlike other anti-angiogenic drugs, it appears to decrease the prevalence of CD206-positive M2-type TAMs in primary tumor (Fig. 6) and metastatic lesions in the kidney (Additional file 4: Figure S4). We are currently exploring whether this effect is due to scVEGF/^177^Lu-induced systemic changes or to specific alterations in tumor vasculature.

## Conclusions

We have demonstrated that targeting VEGF receptors with a single well-tolerated dose of a novel radiopharmaceutical, scVEGF/^177^Lu, delays mortality and inhibits growth of metastatic lesions initiated by intracardiac injection of mouse breast carcinoma cells in immunocompetent mice. A single injection of scVEGF/^177^Lu in combination with a low-dose Doxil blocks tumor growth in an orthotopic human breast carcinoma model. In addition to depleting tumor vasculature of VEGFR-2 overexpressing cells, scVEGF/^177^Lu induced a sustainable decrease in tumor prevalence of pro-tumorigenic/pro-angiogenic M2-type macrophages. Taken together, these findings support further development of targeted scVEGF/^177^Lu radiopharmaceutical.

## Additional files

Additional file 1: Figure S1. Representative image of mouse with 4T1luc bone metastatic lesions. CT and high resolution MicroCT of bone lesion areas show significant pitting and large areas of osteolysis. (PDF 118 kb) Additional file 2: Figure S2. Enlarged high resolution MicroCT images of shoulder area from Additional file 1: Figure S1. MicroCT shows significant pitting and large areas of osteolysis around joints. (PDF 46 kb) Additional file 3: Figure S3. Treatment effects on VEGFR-2 prevalence in tumor. Images of VEGFR-2 immunostaining were captured with 5x objective. For each group, VEGFR-2 prevalence was calculated for 13-14 microscopic fields on immunostained cryosections prepared from tumors harvested from 2-3 mice. (PDF 90 kb) Additional file 4: Figure S4. Rapid declining in VEGFR-2 prevalence in response to scVEGF/^177^ Lu treatment. The difference relative to control was statistically significant (p=0.0001) from Day 1. No statistically significant difference between VEGFR-2 prevalence at days 3 to 15 after treatment. The number of analyzed microscopic fields (N) on cryosections from different mouse tumors (n) for each time point was as following: N/n: Control – 19/4, D1 – 28/6, D3 – 24/5, D5 – 25/6, D8 – 32/6, D11 – 27/6, D15 – 27/6. (PDF 69 kb) Additional file 5: Figure S5. Immunostaining for VEGFR-2 (red) and CD206 (green) on cryosections of 4T1luc kidney metastatic lesions from control (A) and scVEGF/^177^ Lu treated (B) Balb/c mice. 40x objective. (PDF 80 kb)
