# Supplementary material for: Targeted scVEGF/177Lu radiopharmaceutical inhibits growth of metastases and can be effectively combined with chemotherapy
Source: EJNMMI Res. 2016 Jan 16;6:4. doi: 10.1186/s13550-016-0163-1 (PMC4715132; doi:10.1186/s13550-016-0163-1)
Supplement: Additional file 1: Figure S1. — Representative image of mouse with 4T1luc bone metastatic lesions. CT and high resolution MicroCT of bone lesion areas show significant pitting and large areas of osteolysis. (PDF 118 kb) [file 13550_2016_163_MOESM1_ESM.pdf]

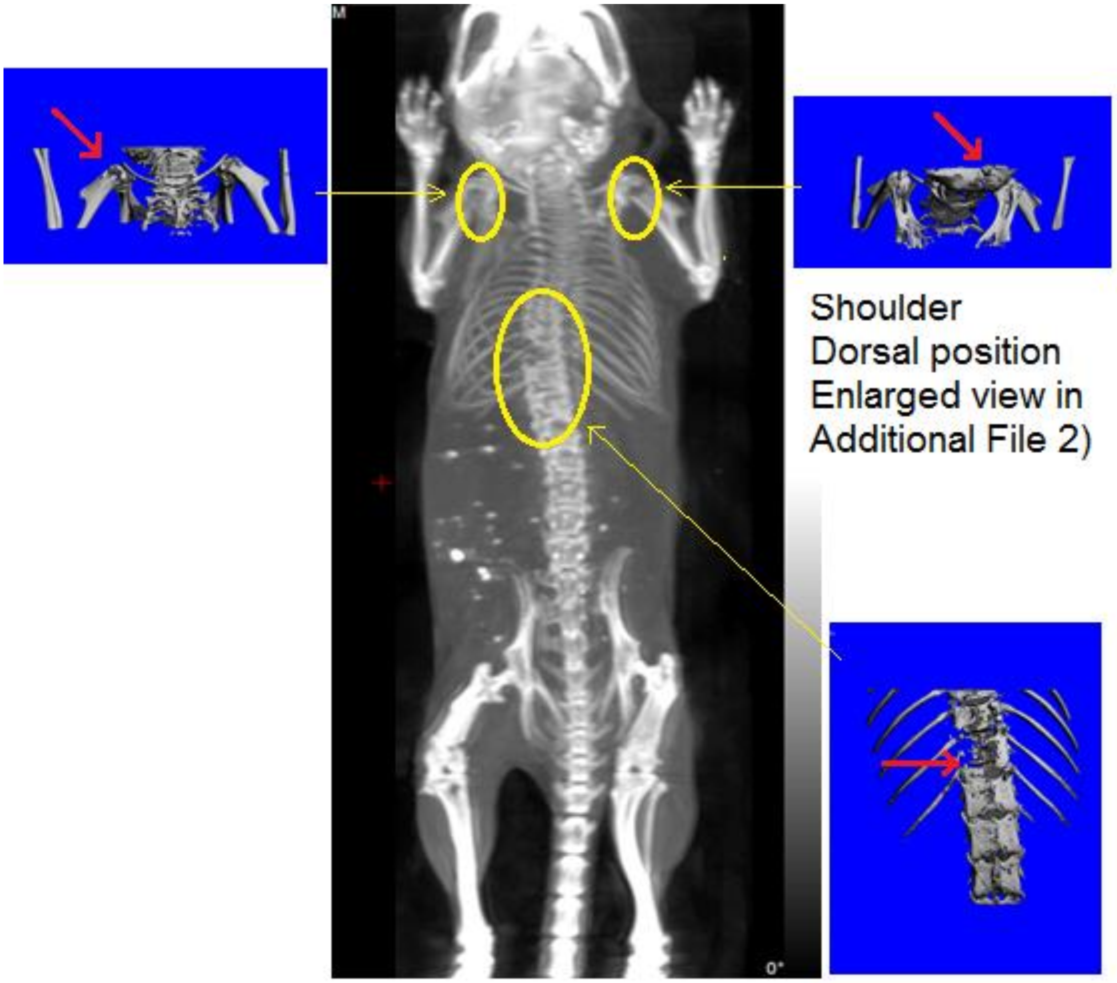

Additional File 1. Representative image of mouse with 4T1luc bone metastatic lesions. CT and high resolution MicroCT of bone lesion areas show significant pitting and large areas of osteolysis.
