# Supplementary material for: Targeted scVEGF/177Lu radiopharmaceutical inhibits growth of metastases and can be effectively combined with chemotherapy
Source: EJNMMI Res. 2016 Jan 16;6:4. doi: 10.1186/s13550-016-0163-1 (PMC4715132; doi:10.1186/s13550-016-0163-1)
Supplement: Additional file 2: Figure S2. — Enlarged high resolution MicroCT images of shoulder area from Additional file 1: Figure S1. MicroCT shows significant pitting and large areas of osteolysis around joints. (PDF 46 kb) [file 13550_2016_163_MOESM2_ESM.pdf]

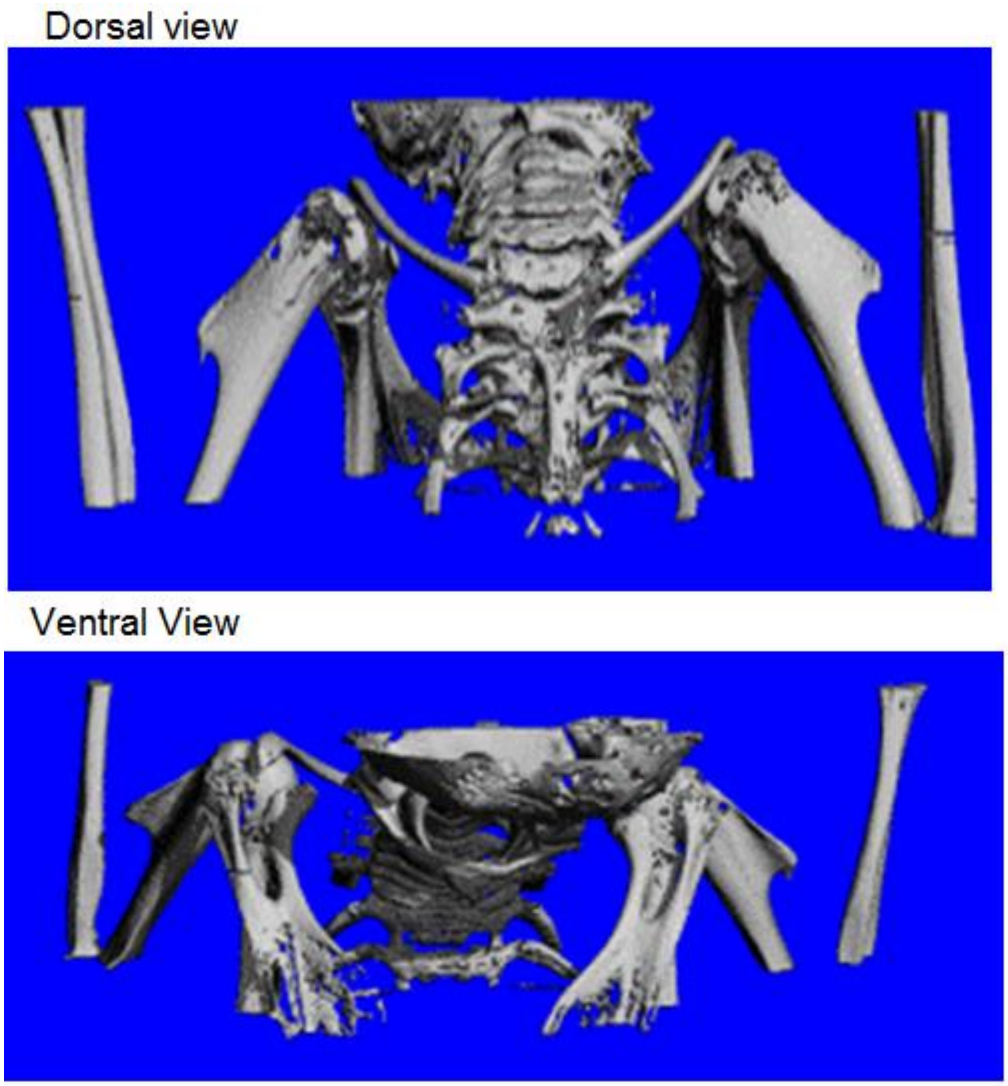

Additional File 2. Enlarged high resolution MicroCT images of shoulder area from Supplementary Figure 1 shows significant pitting and large areas of osteolysis around joints.
