# Supplementary material for: Targeted scVEGF/177Lu radiopharmaceutical inhibits growth of metastases and can be effectively combined with chemotherapy
Source: EJNMMI Res. 2016 Jan 16;6:4. doi: 10.1186/s13550-016-0163-1 (PMC4715132; doi:10.1186/s13550-016-0163-1)
Supplement: Additional file 3: Figure S3. — Treatment effects on VEGFR-2 prevalence in tumor. Images of VEGFR-2 immunostaining were captured with 5x objective. For each group, VEGFR-2 prevalence was calculated for 13-14 microscopic fields on immunostained cryosections prepared from tumors harvested from 2-3 mice. (PDF 90 kb) [file 13550_2016_163_MOESM3_ESM.pdf]

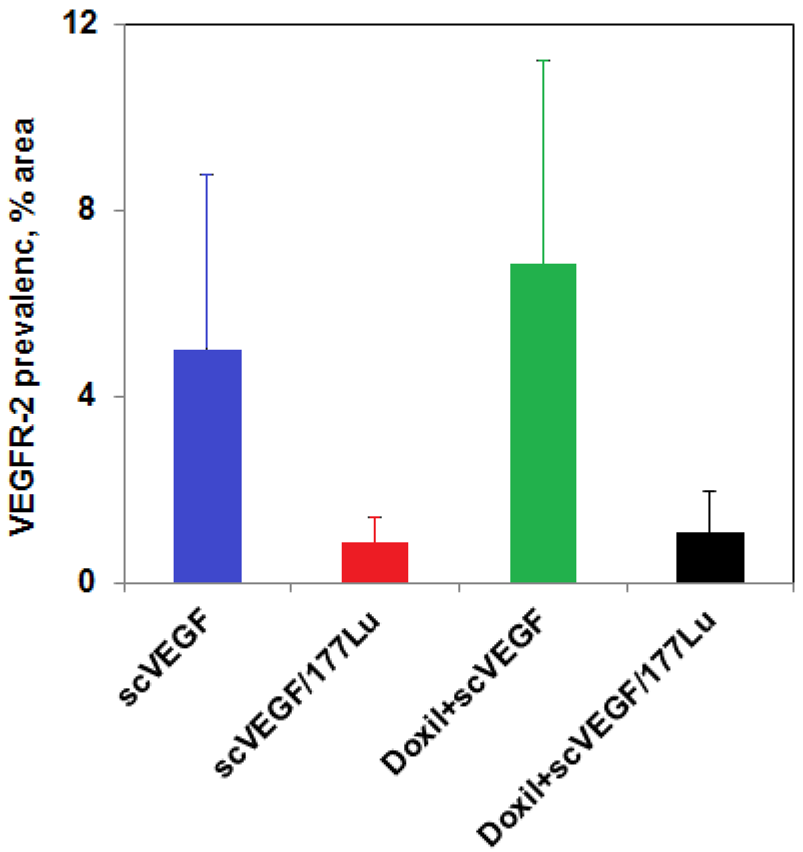

Additional File 3. Treatment effects on VEGFR-2 prevalence in tumor. Images of VEGFR-2 immunostaining were captured with 5x objective. For each group, VEGFR-2 prevalence was calculated for 13-14 microscopic fields on immunostained cryosections prepared from tumors harvested from 2-3 mice.
