# Supplementary material for: Targeted scVEGF/177Lu radiopharmaceutical inhibits growth of metastases and can be effectively combined with chemotherapy
Source: EJNMMI Res. 2016 Jan 16;6:4. doi: 10.1186/s13550-016-0163-1 (PMC4715132; doi:10.1186/s13550-016-0163-1)
Supplement: Additional file 4: Figure S4. — Rapid declining in VEGFR-2 prevalence in response to scVEGF/177 Lu treatment. The difference relative to control was statistically significant (p=0.0001) from Day 1. No statistically significant difference between VEGFR-2 prevalence at days 3 to 15 after treatment. The number of analyzed microscopic fields (N) on cryosections from different mouse tumors (n) for each time point was as following: N/n: Control – 19/4, D1 – 28/6, D3 – 24/5, D5 – 25/6, D8 – 32/6, D11 – 27/6, D15 – 27/6. (PDF 69 kb) [file 13550_2016_163_MOESM4_ESM.pdf]

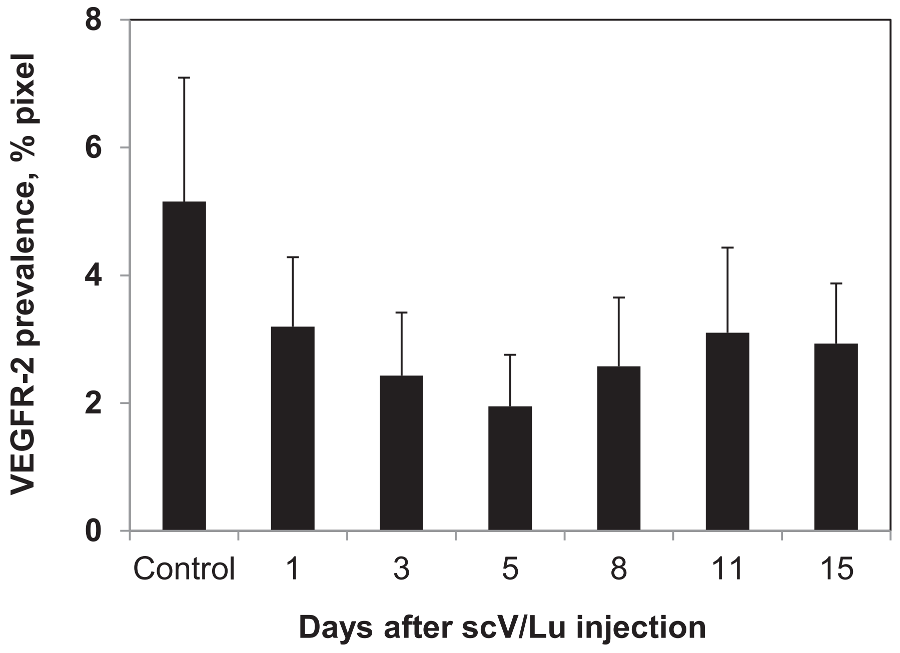

Additional File 4. Rapid declining in VEGFR-2 prevalence in response to scVEGF/177Lu treatment. The difference relative to control was statistically significant (p=0.0001) from Day 1. No statistically significant difference between VEGFR-2 prevalence at days 3 to 15 after treatment. The number of analyzed microscopic fields (N) on cryosections from different mouse tumors (n) for each time point was as following: N/n: Control – 19/4, D1 – 28/6, D3 – 24/5, D5 – 25/6, D8 – 32/6, D11 – 27/6, D15 – 27/6

Treatment effects on VEGFR-2 prevalence in tumor. Images of VEGFR-2 immunostaining were captured with 5x objective. For each group, VEGFR-2 prevalence was calculated for 13-14 microscopic fields on immunostained cryosections prepared from tumors harvested from 2-3 mice.

.
