# Supplementary material for: Targeted scVEGF/177Lu radiopharmaceutical inhibits growth of metastases and can be effectively combined with chemotherapy
Source: EJNMMI Res. 2016 Jan 16;6:4. doi: 10.1186/s13550-016-0163-1 (PMC4715132; doi:10.1186/s13550-016-0163-1)
Supplement: Additional file 5: Figure S5. — Immunostaining for VEGFR-2 (red) and CD206 (green) on cryosections of 4T1luc kidney metastatic lesions from control (A) and scVEGF/177 Lu treated (B) Balb/c mice. 40x objective. (PDF 80 kb) [file 13550_2016_163_MOESM5_ESM.pdf]

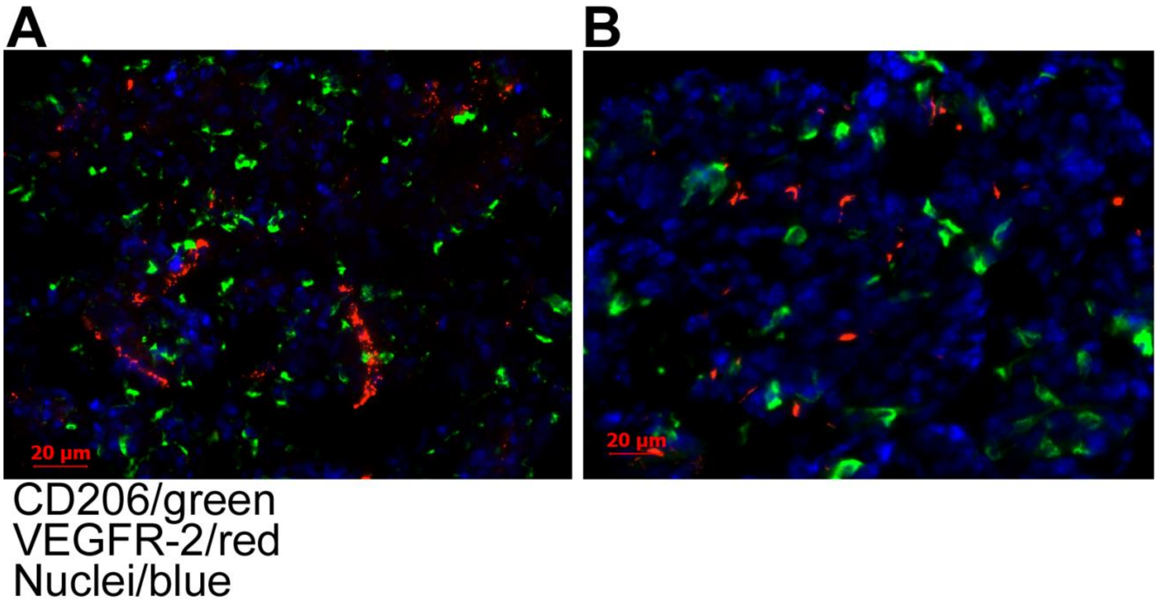

Additional File 5. Immunostaining for VEGFR-2 (red) and CD206 (green) on cryosections of 4T1luc kidney metastatic lesions from control (**A**) and scVEGF/177Lu treated (**B**) Balb/c mice. 40x objective.
